# Congenital Bullous Syphilis: A Case Report from Italy and a Comprehensive Literature Review

**DOI:** 10.3390/medicina61010158

**Published:** 2025-01-18

**Authors:** Edoardo Cammarata, Elia Esposto, Nunzia Di Cristo, Chiara Airoldi, Elena Bernascone, Valentina Burzio, Paola Savoia

**Affiliations:** 1 SCDU Dermatologia, Azienda Ospedaliero-Universitaria Maggiore della Carità, 28100 Novara, Italy; edoardo.cammarata@maggioreosp.novara.it (E.C.); elia.esposto@maggioreosp.novara.it (E.E.); dicristo.nunzia1993@gmail.com (N.D.C.); 2 Department of Translational Medicine, University of Eastern Piedmont, 28100 Novara, Italy; chiara.airoldi@uniupo.it; 3 Pediatric and Neonatal Intesive Care Unit, Azienda Ospedaliero-Universitaria Maggiore della Carità, 28100 Novara, Italy; elena.bernascone@maggioreosp.novara.it (E.B.); valentina.burzio@maggioreosp.novara.it (V.B.); 4 Division of Pediatrics, Azienda Ospedaliero-Universitaria Maggiore della Carità, 28100 Novara, Italy; 5 Department of Health Science, University of Eastern Piedmont, 28100 Novara, Italy

**Keywords:** congenital bullous syphilis, pemphigus syphiliticus, secondary syphilis, bullous lesions, dermatological manifestations, differential diagnosis

## Abstract

*Background and Objectives:* Congenital syphilis remains a significant global health concern, with severe morbidity and mortality if undiagnosed and untreated. Although many infants appear asymptomatic at birth, subtle clinical signs—including bullous lesions (congenital bullous syphilis, also known as pemphigus syphiliticus)—may facilitate early detection. Recognizing this rare manifestation is crucial for timely intervention, reducing serious outcomes. *Materials and Methods:* We systematically reviewed Medline (PubMed), Embase, and the Cochrane Central Register of Controlled Trials from inception to December 2024 for cases of congenital bullous syphilis, also known as pemphigus syphiliticus. We extracted demographic, clinical, laboratory, radiological, treatment, and outcome data. Additionally, we included clinical information from a newly documented case of congenital bullous syphilis managed in our center. *Results*: Twenty-four cases of congenital syphilis with bullous lesions were identified, twenty with sufficient detail for analysis. Patients presented three distinct clinical patterns: confined palmoplantar lesions, acrally distributed lesions, and diffuse bullous-erosive involvement. Despite variable severity, cutaneous manifestations provided a key diagnostic clue. Nontreponemal and treponemal serologic tests were central to diagnosis, supported by maternal screening and imaging. Intravenous penicillin G was the most frequently employed therapy. While most infants achieved remission, severe respiratory involvement was associated with mortality. Our new case aligned with these findings, demonstrating full resolution after appropriate antibiotic therapy. *Conclusions:* Bullous syphilis, though rare, is an important early sign of congenital syphilis. Prompt recognition and diagnosis—enabled by diligent maternal screening, targeted neonatal testing, and careful clinical examination—are essential to initiate timely penicillin therapy and prevent severe complications or death. This review underscores the need for heightened clinical vigilance and adherence to established guidelines for syphilis screening and treatment during pregnancy, ultimately improving neonatal outcomes.

## 1. Introduction

Congenital syphilis is a multisystemic infection caused by Treponema pallidum, acquired through vertical transmission either transplacentally or during passage through the birth canal. In 2022, reported national incidences of congenital syphilis within European Union member states ranged from 0 to 42.4 cases per 100,000 live births, with the highest rate observed in Bulgaria. Although the incidence has fluctuated, a general upward trend has emerged in recent years [1]. In Italy, during the five-year period from 2018 to 2022, the incidence varied between 0.2 and 1.6 cases per 100,000 live births [1].

In pregnant women with untreated early syphilis, vertical transmission to the fetus occurs in the majority of cases (70–100%), commonly after the 28th week of gestation. Prompt maternal treatment prior to this gestational milestone typically prevents fetal involvement. Although many infected newborns are asymptomatic at birth, complications such as prematurity, low birth weight, and even stillbirth—occurring in up to one-third of cases—are frequently observed [1,2,3].

Congenital syphilis often presents differently than in adults. Infected neonates experience direct spirochetemia with the rapid dissemination of T. pallidum to multiple organ systems [2]. Clinical findings at birth vary widely but commonly include hepatosplenomegaly, skeletal abnormalities (osteochondritis and periostitis), jaundice, lymphadenopathy, nasal discharge (luetic coryza), skin rashes, anemia, and thrombocytopenia [2].

Typical skin manifestations consist of diffuse, red-to-brown maculopapular lesions frequently involving the back, buttocks, posterior thighs, and, characteristically, the palms and soles. Over time, these lesions may progress to desquamation and crusting. A bullous variant, known as syphilitic pemphigus or congenital bullous syphilis, is a rare, under-recognized form of an infrequent disease—especially in Europe and North America—which requires rapid treatment [2,4]. It represents a diagnostic challenge to both pediatricians and dermatologists because of the wide differential diagnosis that includes several bullous dermatoses: bullous impetigo/staphylococcal scalded skin syndrome, bullous congenital ichthyosiform erythroderma, neonatal pemphigus, etc.

This paper describes a newly diagnosed case of congenital bullous syphilis at an Italian hospital and presents a comprehensive review of the available literature.

## 2. Materials and Methods

### 2.1. Literature Search Strategy

A comprehensive systematic search was conducted in Medline (PubMed), Embase, and the Cochrane Central Register of Controlled Trials from their respective inceptions up to November 2024. The search strategy incorporated relevant Medical Subject Headings (MeSH) and keywords, including “pemphigus syphiliticus” and “congenital bullous syphilis”. These terms guided the identification of articles that addressed the primary objectives of this review, specifically the demographic and clinical features of congenital bullous syphilis, diagnostic approaches, therapeutic interventions, and patient outcomes.

**Figure 1:**
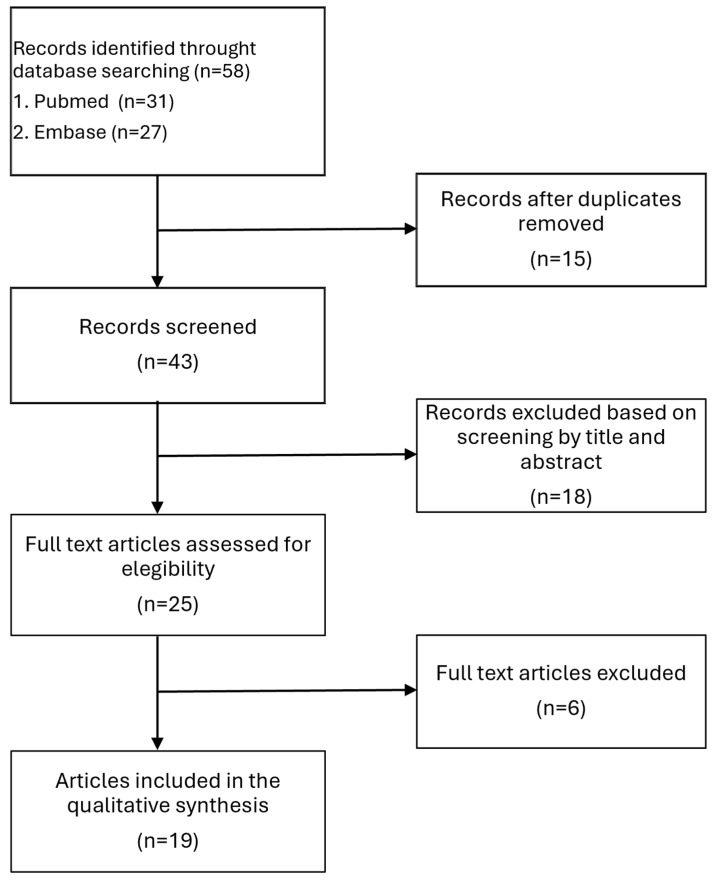
Flow diagram showing the identification of eligible articles.

Two independent reviewers (EC and NDC) screened all retrieved citations. When information in the abstracts was insufficient to determine eligibility, the full texts were examined to ensure that articles met the predefined inclusion and exclusion criteria. Any disagreements between the reviewers were resolved through discussion and consensus, thus maintaining a consistent and transparent selection process (Figure 1). Additionally, the clinical data from the authors’ newly documented case were integrated into the overall dataset, further enhancing the robustness of the pooled analysis and providing a richer understanding of this rare condition.

### 2.2. Statistical Analysis

Descriptive statistics of the subjects included were reported. Categorical variables were summarized using absolute and relative frequencies while, for numerical ones, the mean and standard deviation both with the median and interquartile range were reported. As each study contributed to the overall design with only one subject (case report), estimates were performed without pooling the results. All the analyses were performed using SAS 9.4 and R (2023 version).

## 3. Results

### 3.1. Characteristics of Patients and Infection

From 1950 through December 2024, a total of 24 patients with congenital syphilis were diagnosed and reported in the literature across multiple countries, with 20 cases, including our report, providing sufficient clinical detail for inclusion in this review [4,5,6,7,8,9,10,11,12,13,14,15,16,17,18,19,20,21,22,23,24,25,26]. These cases span a wide geographic range and reflect the ongoing global prevalence of syphilis over several decades, with a noted increase in reporting in recent years. Key demographic, clinical, radiological, and laboratory findings are summarized in Table 1.

Gender distribution was balanced, with 10 female (52.63%) and 9 (47.37%) male patients. The most common initial presentation was a bullous eruption affecting the palms and/or soles (the so-called “palmoplantar” pattern), observed in 11 patients (57.89%). The second most common pattern was diffuse involvement, noted in eight patients (42.11%), typically affecting acral areas as well as other body sites. These lesions most frequently appeared at birth (n = 17, 85.0%), with only one at 25 days, one at 60 days, and one at 3 months. Moreover, 10 (52.63%) times were observed in preterm infants. The predominant associated cutaneous manifestations included erythema (n = 16, 80.0%), desquamation (n = 15, 75.0%), and erosions (n = 11, 55.0%).

Early congenital syphilis frequently presented with hepatosplenomegaly (n = 14, 70.0%), respiratory distress (n = 8, 40.0%), and luetic coryza (n = 4, 20.0%). These findings often coexist with congenital bullous syphilis. Additionally, osteitis, periostitis, and metaphyseal destruction were reported in 42.11% (n = 8) of patients. Laboratory abnormalities commonly included thrombocytopenia, anemia and both leukocytosis and elevated transaminases, with respective frequencies of 42.11%, 21.05%, and 15.79%.

### 3.2. Diagnosis, Treatment of Infection, and Outcomes

Diagnosis was established through a combination of clinical evaluation, radiologic assessment, and laboratory investigations. Nontreponemal tests, including the Rapid Plasma Reagin (RPR) and the Venereal Disease Research Laboratory (VDRL) test, were the most frequently performed serologic procedures, conducted in 90.0% of mothers and 100% of newborns. Treponemal tests, such as the Treponema pallidum Hemagglutination Assay (TPHA) or Treponema pallidum Particle Agglutination Assay (TPPA), were performed in 60.0% (n = 12) of maternal and 52.63% (n = 10) neonatal cases, respectively. Analysis of cerebrospinal fluid (CSF) was undertaken in 47.06% (n = 9) of newborns, with the VDRL as the most commonly reported CSF test (36.84%, n = 7). Additional assays, including the Fluorescent Treponemal Antibody Absorption (FTA-ABS) test and ELISA for IgM antibodies, were also frequently employed.

Penicillin G represented the most commonly prescribed specific treatment (88.89%), followed by intramuscular penicillin (11.1%). In some instances, empirical antibiotic therapy was initiated before confirmation of the diagnosis. Penicillin dosing regimens varied considerably among the reported cases, ranging from 100,000 U/kg IV for 14 days to 2,000,000 U/kg/day IV for 10 days. Overall, most patients achieved complete remission (80.0%). However, one patient (5.0%) developed neurosensory deafness as a complication, and three patients (15.0%) unfortunately died, with respiratory insufficiency and multiorgan failure cited as the leading causes of mortality. A detailed summary of these data is provided in Table 2.

### 3.3. Our Case

We present the case of a female newborn delivered at 35 + 3 weeks of gestational age (GA) by urgent cesarean section due to nonreassuring fetal heart tracings. At birth, the infant’s weight was appropriate for GA (2270 g). The amniotic fluid was Grade III meconium-stained, and the Apgar scores were one at both 1 and 5 min. Initial examination revealed hepatosplenomegaly, mild axial hypotonia, and reduced mobility of the right wrist and hand with a weak grasp, although archaic reflexes were normal, and lymph nodes were not palpable.

Within the first hour of life, the newborn developed respiratory distress requiring escalating respiratory support, initially via high-flow nasal cannula (HFNC) and subsequently non-invasive positive pressure ventilation (NIPPV) with an FiO_2_ up to 40%. At six hours of life, due to further clinical deterioration, the infant was nasotracheally intubated and administered surfactant. Blood tests indicated hemolytic anemia and thrombocytopenia, necessitating platelet and packed red blood cell transfusions.

**Figure 2:**
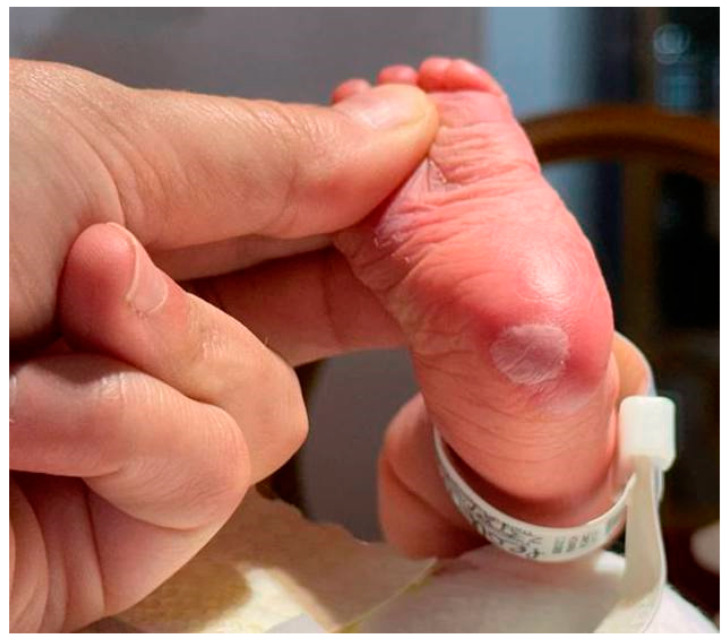
Single plantar erythematous bullous lesion present since birth.

Dermatologic examination revealed petechiae over the trunk at the proximal regions of the upper and lower limbs, two plantar bullous lesions measuring approximately 0.5 to 1.5 cm, and a small de-epithelialized lenticular area on the back of the left hand (Figure 2). Given the presence of hepatosplenomegaly, thrombocytopenia, and acral bullous lesions, both maternal and neonatal RPR and TPHA tests were obtained. The neonate’s RPR was positive at 1:1, and the maternal RPR was also positive at 1:1. The neonatal TPHA titer was 1:2560, mirroring the maternal titer of 1:2560. The newborn tested negative for IgM antibodies, while the mother tested positive. HIV, HCV, and HBV serologies were negative in both. Blood cultures ruled out bacterial infection, including staphylococcal involvement. Although the mother’s first-trimester screening was normal, she did not attend the recommended clinical and serological follow-up in the third trimester.

A diagnosis of congenital bullous syphilis was established. Treatment with aqueous crystalline penicillin G at 100,000 units/kg/day IV was initiated, given as 50,000 units/kg per dose every 12 h for the first seven days of life, then every eight hours thereafter for a total of ten days.

Five hours after the first penicillin dose, the infant developed fever (skin temperature 38 °C) and hypotension resistant to fluid boluses, necessitating a norepinephrine infusion. This therapy was discontinued on the fourth day after hemodynamic stabilization, likely representing a Jarisch–Herxheimer reaction.

Neurological evaluation, including cerebrospinal fluid analysis, was normal. Upper limb radiographs showed areas of rarefaction consistent with luetic metaphysitis. Abdominal and cardiac ultrasound, as well as otolaryngological and ophthalmological examinations, revealed no abnormalities. The ulcerated skin lesions resolved by the seventh day of treatment. The infant was discharged in stable condition following the complete course of antibiotic therapy.

## 4. Discussion

Congenital syphilis is a preventable infection that, if untreated, can lead to severe acute and long-term complications. Approximately one-third of affected fetuses experience miscarriages or stillbirths, and, during the perinatal period, morbidity and mortality rates are estimated at 33.6–40% and 6.5–10%, respectively [27,28,29,30]. Notably, up to two-thirds of newborns with congenital syphilis may be asymptomatic at birth, with low birth weight often the only initial clinical sign. Although cutaneous manifestations in congenital syphilis are typically nonspecific—most commonly, a diffuse erythematous, desquamative rash—less frequently reported bullous and erosive lesions can serve as critical diagnostic clues [1,2,3,4]. Such findings significantly narrow the differential diagnosis to a select group of conditions, including acral peeling syndrome, congenital epidermolysis bullosa, bullous impetigo/staphylococcal scalded skin syndrome, neonatal pemphigus, congenital bullous syphilis, and erythema multiforme [4,5].

Distinguishing these entities demands thorough clinical evaluations, detailed maternal and neonatal histories, appropriate serological tests, and radiographic imaging. Congenital bullous syphilis is distinguished by flaccid bullae and erosions, often with minimal or absent peripheral erythema. In this review, we identified three distinct clinical phenotypes of congenital bullous syphilis: a confined palmoplantar pattern, an acral distribution, and a more diffuse rash associated with severe bullous and erosive lesions. Recognizing these variants is crucial for guiding early diagnosis and ensuring timely, appropriate intervention.

The early detection and management of congenital syphilis are contingent upon identifying active infection in pregnant women. Routine screening with a nontreponemal test (RPR or VDRL) is recommended during the first and third trimesters. Neonates born to mothers with suspected or confirmed syphilis should also be tested. A neonatal nontreponemal titer fourfold higher than the maternal titer is highly indicative of congenital syphilis [14]. Treponemal IgG antibodies can persist in the neonate due to passive transfer from the mother and remain for up to 18 months, rendering them less useful for initial diagnosis. In contrast, the detection of IgM antibodies, which are not transferred from the mother, can support the diagnosis; however, a negative IgM test at birth does not rule out congenital syphilis [31].

The management of congenital syphilis follows CDC guidelines [3], which stratify treatment intensity based on diagnostic certainty. Confirmed or highly probable congenital syphilis, as well as possible congenital syphilis, typically warrants intravenous aqueous crystalline penicillin G therapy (100,000–150,000 units/kg/day for 10 days). Procaine penicillin G or, in certain circumstances, benzathine penicillin G may be considered in possible or less likely congenital syphilis.

Our findings suggest that the extent of cutaneous involvement may not correlate directly with patient prognosis. Instead, severe pulmonary involvement emerged as a leading cause of mortality in the cases examined. Among the three fatal cases reported in the literature, two did not receive intravenous penicillin G (instead receiving intramuscular forms), and, in the third case, treatment details were not documented. These observations highlight the importance of both the appropriateness and the route of therapy.

## 5. Conclusions

In this study, we present a newly described case of congenital syphilis and provide a comprehensive review of the existing literature. This work underscores the significance of recognizing congenital bullous syphilis as an early, albeit rare, marker of the disease. Dermatologists, pediatricians, and other frontline clinicians should maintain a high index of suspicion for congenital syphilis when encountering neonatal bullous lesions, ensuring early diagnosis and prompt treatment to prevent potentially fatal sequelae.

## Figures and Tables

**Table 1:** Characteristics of patients and infection.

Variables	N (%)
Described cases	N = 20
Country of origin	N = 20
Asia	10 (50.0%)
Europe	4 (20.0%)
America	6 (30.0%)
Sex	N = 19
Female	10 (52.63%)
Male	9 (47.37%)
Present at birth	N = 20
Yes	17 (85.0%)
Until 2 years of age	3 (15.0%)
Pregnancy	N = 19
Full-term	9 (47.37%)
Pre-term	10 (52.63%)
Erosive-bullous eruption: forms of presentation	N = 19
Palmoplantar	11 (57.89%)
Diffuse	8 (42.11%)
Concomitant skin manifestations	N = 20
Erythema	16 (80.0%)
Desquamation	15 (75.0%)
Erosions	11 (55.0%)
Pustules	3 (15.0%)
Condyloma lata	2 (10.0%)
Others (Petechiae, Acrocyanosis, Cheilitis)	3 (15.0%)
Concomitant systemic manifestations	N = 20
Hepato-splenomegaly	14 (70.0%)
Respiratory distress	8 (40.0%)
Luetic coryza (nasal discharge)	4 (20.0%)
Others (Jarisch–Herxheimer, Parrot pseudoparalysis, sepsis, cataracts, cyanosis, etc.)	12 (60.0%)
Radiographic manifestations	N = 19
Osteitis—metaphyseal periostitis	8 (42.11%)
None	11 (57.89%)
Laboratory	N = 19
Thrombocytopenia (N = 17)	8 (42.11%)
Anemia	4 (21.05%)
Leucocytosis	3 (15.79%)
Elevated transaminases	3 (15.79%)
Acidosis	2 (10.53%)
Hyperbilirubinemia	2 (10.53%)
Cholestasis	1 (5.26%)

**Table 2:** Diagnosis, treatment of infection, and outcome.

Variables	N (%)
Maternal diagnosis	N = 20
RPR/VDRL	18 (90.0%)
TPPA/TPHA	12 (60.0%)
Others	4 (20.0%)
Newborn diagnosis	N = 19
RPR/VDRL	18 (94.12%)
TPPA/TPHA	10 (52.63%)
CSF—(VDRL)	9 (47.06%)—7 (36.84%)
Specific antibodies	6 (35.29%)
Skin biopsy	4 (23.53%)
Systemically treated patients	N = 18
Penicillin G IV	16 (88.89%)
Penicillin IM	2 (11.1%)
Empiric antibiotic therapy	3 (16.66%)
Outcome	N = 20
Complete Healing	16 (80.0%)
Death	3 (15.0%)
Neurosensory deafness	1 (5.0%)

## Data Availability

The original contributions presented in this study are included in the article. Further inquiries can be directed to the corresponding author.
